# Next-Generation Theranostic Agents Based on Polyelectrolyte Microcapsules Encoded with Semiconductor Nanocrystals: Development and Functional Characterization

**DOI:** 10.1186/s11671-018-2447-z

**Published:** 2018-01-25

**Authors:** Galina Nifontova, Maria Zvaigzne, Maria Baryshnikova, Evgeny Korostylev, Fernanda Ramos-Gomes, Frauke Alves, Igor Nabiev, Alyona Sukhanova

**Affiliations:** 1 Laboratory of Nano-Bioengineering, National Research Nuclear University MEPhI (Moscow Engineering Physics Institute), Kashirskoye Shosse 31, Moscow, Russian Federation 115409; 2 N.N. Blokhin National Medical Research Center of Oncology, Kashirskoye Shosse 24, Moscow, Russian Federation 115478; 3 Moscow Institute of Physics and Technology (State University), Institutskiy per. 9, Dolgoprudny, Moscow Region Russian Federation 141701; 4 Translational Molecular Imaging, Max-Planck-Institute of Experimental Medicine, Hermann-Rein-Str. 3, 37075 Göttingen, Germany; 5 Clinic of Haematology and Medical Oncology, University Medical Center Göttingen, Robert-Koch-Str. 40, 37075 Göttingen, Germany; 6 Laboratoire de Recherche en Nanosciences (LRN-EA4682), Université de Reims Champagne-Ardenne, rue Cognacq Jay 51, 51095 Reims, France

**Keywords:** Semiconductor nanocrystals, Polyelectrolyte microcapsules, Encoding, Theranostic agents

## Abstract

Fabrication of polyelectrolyte microcapsules and their use as carriers of drugs, fluorescent labels, and metal nanoparticles is a promising approach to designing theranostic agents. Semiconductor quantum dots (QDs) are characterized by extremely high brightness and photostability that make them attractive fluorescent labels for visualization of intracellular penetration and delivery of such microcapsules. Here, we describe an approach to design, fabricate, and characterize physico-chemical and functional properties of polyelectrolyte microcapsules encoded with water-solubilized and stabilized with three-functional polyethylene glycol derivatives core/shell QDs. Developed microcapsules were characterized by dynamic light scattering, electrophoretic mobility, scanning electronic microscopy, and fluorescence and confocal microscopy approaches, providing exact data on their size distribution, surface charge, morphological, and optical characteristics. The fluorescence lifetimes of the QD-encoded microcapsules were also measured, and their dependence on time after preparation of the microcapsules was evaluated. The optimal content of QDs used for encoding procedure providing the optimal fluorescence properties of the encoded microcapsules was determined. Finally, the intracellular microcapsule uptake by murine macrophages was demonstrated, thus confirming the possibility of efficient use of developed system for live cell imaging and visualization of microcapsule transportation and delivery within the living cells.

**Electronic supplementary material:**

The online version of this article (10.1186/s11671-018-2447-z) contains supplementary material, which is available to authorized users.

## Background

The use of polyelectrolyte microcapsules as vehicles for targeted delivery and controlled release of drugs and contrast agents and as fluorescent probes for in vitro and in vivo imaging is a promising line of research in translational medicine and personalized approach to diagnosis and treatment of various human diseases [1–4].

Development of theranostic agents combining the functions of drugs and tools for imaging of biomarkers allowing early diagnosis of various diseases is an important task in the field of designing drug delivery systems [5, 6]. Systems based on polyelectrolyte microcapsules are promising candidates for combining both functions. The conditions of their fabrication allow the incorporation of biologically active substances, metal nanoparticles, fluorescent labels, etc. into the microcapsules [7–9]. Additional file 1: Figure S1 schematically shows a typical theranostic agent based on polyelectrolyte microcapsules.

One of effective methods for obtaining polyelectrolyte microcapsules consists in successive application of oppositely charged polymer layers onto a substrate of spherical or other shape, which is removed afterwards [10, 11]. Interaction of the oppositely charged polycation and polyanion at specified pH, ionic strength, and temperature of the solution and polarity of the solvent results in an interpolymer complex in the form of a membrane or shell coating the substrate [12–14].

The factors listed above also affect the morphology of the resultant microcapsules, including their porosity and shape and the integrity of the wall. For example, an increase in the ionic strength or pH of the environment of the polyelectrolyte microcapsules facilitates conformational changes or protonation/deprotonation of the polyelectrolytes forming the capsule wall. This, in turn, leads to its deformation and increase in the porosity even to the degree of the loss of structural integrity and transition to the “open” state followed by release of the inner contents of the capsules and the components embedded in their walls into the microenvironment [15, 16]. These properties make polyelectrolyte microcapsules good candidates for the role of stimulus-sensitive delivery systems and a promising basis for designing theranostic agents [2, 17, 18].

Quantum dots (QDs) are fluorescent semiconductor nanocrystals 2–10 nm in diameter characterized by a wide absorption spectrum and a narrow, symmetrical fluorescence spectrum. This allows QDs with different fluorescence maxima to be excited from a single radiation source, offering the possibility of their wide use as fluorophores, especially for multiplexed imaging [19, 20]. A high photostability and bright fluorescence of QDs determine their advantage over standard organic fluorophores in detection applications [21–24].

Earlier published studies devoted to fluorescent polymeric polyelectrolyte microcapsules development demonstrate one typical approach to classical organic fluorescent dyes or in situ forming carbon dots under hydrothermal carbonization and conversion of dextran to luminescent carbon nanoparticles into polyelectrolyte shell entrapment within the polymeric structure of the primary prepared polyelectrolyte microcapsules. The approach of organic dye entrapment is based on the diffusion of the fluorescein isothiocyanate or rhodamine B, tetramethylrhodamine dyes conjugated with low molecular weight dextrane or bovine serum albumin (BSA) into the porous structure of the membrane formed by polyelectrolytes that results in fluorescent dye charging of the whole structure of the polyelectrolyte microcapsule as in interior hollow and as in the polymeric membrane. The necessity of high thermal treatment in case of carbon dot-encoded microcapsules changes the flexibility of the microcapsule structure and makes it more rigid that is not undesirable in case of pH and ionic strength stimuli-responsive theranostic systems development [25–30].

In this study, we are describing all technological aspects of fabrication of polymeric microcapsules encoded with the highly fluorescent water-soluble QDs, possessing significant colloidal stability, describe their physico-chemical and functional properties and demonstrate their application to live cell imaging and visualization of microcapsules transportation and delivery within the living cells. The data may pave the way to the next step to development of the next generation of theranostic agents based on the multifunctional functionalized microcapsules.

## Experimental

### Solubilization and Characterization of Quantum Dots

CdSe/ZnS core/shell QDs with a fluorescence maximum λ_max_ equal to 590 nm were kindly provided by Dr. Pavel Samokhvalov (Laboratory of Nano-Bioengineering, National Research Nuclear University MEPhI (Moscow Engineering Physics Institute), Moscow, Russia).

Freshly synthesized QDs were coated with trioctylphosphine oxide (TOPO) and were water-insoluble. Their transfer to the water phase was performed by substituting d,l-cysteine for TOPO and subsequently replacing of d,l-cysteine with 12*-*unit PEG derivative containing thiol and carboxyl end groups HS−(PEG)_12_−COOH (Thermo Fisher Scientific, USA) as described earlier [22, 31, 32]. For this purpose, a sample of QDs was dissolved in 800 μl of chloroform, after which 1200 μl of methanol was added, and the mixture was centrifuged for 5 min. The procedure was repeated three times. Then, the QD pellet was resuspended in 800 μl of chloroform. A solution of d,l-cysteine in methanol was added to the suspension with a QD to d,l-cysteine weight ratio of 1:0.13, and the mixture was centrifuged at 16,873*g* for 10 min (Centrifuge 5418, Eppendorf, USA). The QD pellet was washed of excess d,l-cysteine with methanol by means of centrifugation for 3 min at the same speed. The QD pellet was dried in a Concentrator Plus centrifugal concentrator (Eppendorf, USA) for 2 min. The dried QDs were suspended in 650 μl of 0.1 M sodium hydroxide and sonicated for 10 min in an Elma Sonic P30H ultrasound bath (Elma Schmidbauer, Germany). Then, the solution was centrifuged at 5509*g* for 10 min, and the supernatant was filtered through a Millipore filter with a pore size of 0.22 μm (Merck, Germany). The QD content of the samples was determined spectrophotometrically at the wavelength of the first exciton absorption peak.

The obtained water-soluble QD samples were stabilized by adding HS−(PEG)_12_−COOH at a QD to PEG derivative molar ratio of 1:4.6 and incubating the mixture at 2–8 °C for 24–48 h.

### Synthesis of Calcium Carbonate Microparticles

Calcium carbonate microparticles were obtained as described elsewhere [33, 34]. Fifteen ml of a 0.33 M Na_2_CO_3_ (Sigma-Aldrich, Germany) solution was added to 15 ml of a 0.33 M СаСl_2_ (Sigma-Aldrich, Germany) solution while stirring. The reaction mixture was stirred at rates of 250, 500, and 750 rpm on an RCT Basic magnetic stirrer (IKA, Germany) at room temperature for 15 to 60 s. The СаСl_2_ and Na_2_CO_3_ solutions were preliminarily filtered through filters with a pore size of 0.22 μm.

After that, the stirring was stopped, and the reaction mixture was incubated for 10 min. The mixture was washed with MilliQ water by alternatingly resuspending and centrifuging at 452*g* for 5 min using a Centrifuge 5810 (Eppendorf, USA). The obtained microparticles were washed four times. After the final washing, the pellet was dried in an oven at 60 °C for 90 min.

### Preparation of Polyelectrolyte Microcapsules Encoded with Quantum Dots

The microparticles were encoded with QDs using a modified technique of layer-by-layer deposition of oppositely charged polymers [31, 35] and carboxylated water-soluble QDs onto prepared calcium carbonate microparticles, which served as a matrice. The polyelectrolyte layers consisted of pairs of polymers: the polycation poly(allylamine hydrochloride) (PAH) with Mw ≈ 15,000 Da (Sigma-Aldrich, USA) and the polyanion poly(sodium 4-styrenesulfonate) (PSS) with Mw ≈ 70,000 Da (Sigma-Aldrich, USA).

The layers were applied in the following order: СаСО_3_/PAH/PSS/PAH/PSS/PAH/QD-S-(PEG)_12_-COOH/PAH/PSS/PAH/PSS/PAH/PSS.

A sample of dried microparticles was resuspended in 0.5 ml of MilliQ water and sonicated in an ultrasonic bath for 10 min. A 0.5-ml aliquot of 2 mg/ml PAH solution in 0.5 M NaCl was added to the suspension containing 3.7 × 10^8^ calcium carbonate microparticles in MilliQ water. The suspension was sonicated in an ultrasonic bath for 60 s and then incubated for 20 min while stirring. After that, the microparticle suspension was washed of excess polymer by centrifugation at 1054*g* for 5 min followed by resuspension in MilliQ water. The washing of calcium carbonate microparticles after the layering of the polycation was repeated three times. For application of the next (polyanion) layer, the microparticles were preliminarily resuspended in 0.5 ml of MilliQ water; the suspension was mixed with 0.5 ml of a 2 mg/ml PSS solution in 0.5 M NaCl, sonicated in an ultrasonic bath for 60 s, incubated for 20 min while stirring, and then washed of excess polymer as described above.

Five polyelectrolyte layers, the outer layer consisting of PAH, were applied onto the calcium carbonate particles before encoding. From 0.10 to 2.24 mg of QDs was added to the suspension of the microparticles. The mixture was incubated for 80 min while stirring and then washed three times by centrifugation as described above. After that, successive layers of oppositely charged polymers were applied. The encoded microparticles were stored at +  4 °C in the dark.

For obtaining QD-encoded polyelectrolyte microcapsules, the calcium carbonate core was removed from the microparticles. For this purpose, after centrifugation, the pellet of QD-encoded microparticles was resuspended in 2 ml of 0.2 M disodium ethylenediaminetetraacetate (EDTA) (pH 6.5), and the suspension was incubated for 15 min. To guarantee the dissolution of the calcium carbonate core, we repeated this procedure two more times, each time replacing the solution with a fresh aliquot of 0.2 M EDTA (pH 6.5) after 5-min centrifugation of the sample at 2152*g*. Then, the suspension of QD-encoded microcapsules was washed of excess EDTA four times by resuspending in MilliQ water and centrifuging under the conditions specified above. The resultant polyelectrolyte microcapsules were stored at +  4 °C in the dark.

When studying the interaction of the QD-encoded polyelectrolyte microcapsules with cells, we modified the microcapsule surface with BSA (heat shock fraction, protease free, low endotoxin, suitable for cell culture, pH 7, ≥ 98%; Sigma-Aldrich, USA). Briefly, the encoded microparticles with a polycation upper layer were additionally coated with a polyanion polyacrylic acid (PAA) with Mw ≈ 15,000 Da (Sigma-Aldrich, USA), and the core was removed as described above. After the final washing, the microcapsules were dispersed in a 50 mM phosphate buffer solution (pH 7.4) containing 1% of BSA and incubated at + 4 °C in the dark. Before use, the microcapsules were washed of excess BSA with a 50 mM phosphate buffer solution (pH 7.4).

### Characterization of the Quantum Dots, Microparticles, and Polyelectrolyte Microcapsules Encoded with Quantum Dots

#### Size and Charge Study

The hydrodynamic diameter of the solubilized QDs, QD-encoded microparticles coated with polymer shells, and QD-encoded polyelectrolyte microcapsules were determined by the dynamic light scattering method; the surface charge was estimated from their electrophoretic mobility using the Doppler effect by means of a Zetasizer NanoZS (Malvern, UK).

#### Fluorescence Analysis

The fluorescence lifetimes (fluorescent decay kinetics) of the solubilized QDs, QD-encoded microparticles coated with polymer shells, and QD-encoded polyelectrolyte microcapsules were measured at the wavelength of the fluorescence maximum. The second harmonic of an YAG:Nd^3+^ laser with a pulse length of 350 ps and pulse rate of 50 Hz was used as an excitation source. The signal was detected by a photomultiplier detector connected with a DPO 3054 oscillograph (Tektronix, USA) with a time resolution of 2 ns. The suspensions of QD-encoded microparticles and microcapsules were permanently stirred during the measurements by means of a MIXcontrol eco magnetic stirrer (2mag, Germany) to prevent sedimentation of the sample.

#### Estimation of the Encoding Efficiency

The encoding efficiency was estimated from the QD content of the supernatant after the application (adsorption) of QDs onto the microparticle surface. The amount of QDs adsorbed on the microparticle surface (${Q}_{{\mathrm{QD}}_{\mathrm{abs}}}$) was calculated as

$$ {Q}_{{\mathrm{QD}}_{\mathrm{abs}}}={Q}_{{\mathrm{QD}}_0}-{Q}_{{\mathrm{QD}}_i}, $$

where ${Q}_{{\mathrm{QD}}_0}$ is the initial amount of QDs in the aliquot used for encoding, and ${Q}_{{\mathrm{QD}}_i}$ is the amount of QDs in the supernatant of the *i*th sample.

The QD content of samples was determined spectrophotometrically using an Infinite 200 PRO multimode plate reader (Tecan, Switzerland).

#### Scanning Electron Microscopy

Electron microphotographs of the calcium carbonate microparticles were obtained using a JSM-7001F scanning electron microscope (JEOL, Japan) equipped with a Schottky cathode. The powder of dried microparticles was applied onto a conducting carbon adhesive tape and scanned at an averaging of 50, a beam current of 20 pA, and an accelerating voltage of 15–30 kV.

For obtaining microphotographs of the microparticles coated with layers of polyelectrolytes, a drop of a diluted microparticle suspension containing ~ 10^6^ microparticles per 0.5 ml was placed onto a preliminarily purified silicon substrate and dried at room temperature. The resultant samples were scanned at an averaging of 50, a beam current of 20 pA, and an accelerating voltage of 3–30 kV.

#### Fluorescence and Confocal Microscopy

The morphology and size distribution of the QD-encoded microparticles were analyzed by means of fluorescence microscopy using a Carl Zeiss Axio Scope A1 microscope (Carl Zeiss, Germany) equipped with Texas Red fluorescence emission filter; 20% aqueous solution of glycerol was used as a slide mounting media*.*

The samples of QD-encoded microcapsules were also studied using a Leica TCS SP5 confocal laser scanning microscope (Leica Microsystems, Germany) equipped with lasers for excitation 405, 458, 476, 488, 496, 514, 561, and 633 nm and Leica LAS AF software version 2.7.3.9723. The analysis was conducted at the excitation wavelength 488 nm and collecting filters set covering the emission range at 555–620 nm using Leica HCX PL APO CS 63×/1.20 CORR WATER objective. Twenty percent solution of glycerol in PBS buffer pH 7.4 was used as a slide mounting media. The Image J 1.51 s software (USA) was used for image analysis and processing.

### Uptake of Polyelectrolyte Microcapsules Encoded with Quantum Dots by Live Cells in Vitro

The immortalized mouse alveolar macrophage cell line MH-S (ATCC, USA) was maintained in RPMI medium supplemented with 10% FCS, 0.05 mM 2-mercaptoethanol and 2.06 mM glutamine in a humidified atmosphere at 5% CO_2_ and 37 °C. The MH-S cells cultured up to 3×10^6^ cells in 35-mm μ-dishes and 1.2×10^6^ of the QD-encoded polyelectrolyte microcapsules coated with BSA were added to each μ-dishe. The cells were further incubated at 37 °C and 5% CO_2_ for 4 and 24 h respectively. Then, the cell nuclei were counterstained using DRAQ5 fluorescent probe (ex/em wavelengths 646/697 nm, ThermoFisher, USA) during 30 min and afterwards the cell samples were washed and analyzed using Leica TCS SP5 confocal laser scanning microscope (Leica Microsystems, Germany). The images of the cellular uptake of the QD-encoded polyelectrolyte microcapsules were acquired using HCX PL APO CS 63.0 × 1.30 GLYC/OIL, HCX PL APO lambda blue 40.0 × 1.25 OIL. The fluorescence of the QDs was excited at 488 nm and the emission was collected at 555–620 nm, while the fluorescence of the cell nuclei counterstained with DRAQ5 was excited at 633 nm and the emission collected at 650–750 nm.

### Statistical Analysis

The statistical analysis was performed using the MS Office Excel 2007 and Origin Pro 2015 software. All the data are shown as the means and standard deviations using the results from as minimum three independent experiments.

## Results and Discussion

### Synthesis and Characterization of Calcium Carbonate Microparticles

The use of spherical inorganic crystals, in particular, calcium carbonate microspherolites, as a substrate is determined by their biocompatibility, as well as the possibility of their removal in the course of the formation of polyelectrolyte microcapsules without using solvents aggressive for living systems. Calcium carbonate microparticles per se can also be used as a system of drug delivery with modified or prolonged release, serving as a matrix for loading drugs and controlling their release into the microenvironment, i.e., they have multiple potential uses in delivery systems [36–46].

The key factors determining the size and shape of the microcrystals are the rate and duration of stirring and the time of incubation of the reaction mixture [33, 41]. We have experimentally determined the conditions for obtaining calcium carbonate microspherolites with an optimal size distribution. Single CaCO_3_ microparticles have been found to have an almost regular rounded shape. Additional file 1: Figure S2 shows the size distributions of calcium carbonate microparticles obtained at different stirring rates. As seen from these data, the size heterogeneity of the particles increased with increasing stirring rate. If the stirring rate was 250 rpm, the size of the particles obtained ranged from 4.0 to 6.0 μm. In this case, all microparticles in the sample were separate, and their size distribution was close to normal (Additional file 1: Figure S2a). However, if the mixture was stirred at 500 rpm, we observed particles of irregular shape which were aggregations of smaller particles, the mean size of individual particles varying from 2.7 to 5.6 μm (Additional file 1: Figure S2b). At a stirring rate of 750 rpm, the scatter of the particle size was increased. This sample also contained irregularly shaped aggregations, with the mean size of individual microparticles in the range from 3.8 to 5.7 μm (Additional file 1: Figure S2c).

Thus, stirring of the reaction mixture at a rate of 250 rpm made it possible to obtain particles with an optimal size distribution and a nearly regular shape and prevented particle aggregations. Therefore, we estimated the effect of the stirring duration on the microparticle size distribution at this stirring rate (Additional file 1: Figure S3). Stirring of the reaction mixture for 15 s yielded an increased number of larger particles compared to 30-s stirring. An increase in the stirring duration to 60 s had a similar effect. That is, the stirring duration was insufficient in the former case (Additional file 1: Figure S3a) and excessive in the latter case (Additional file 1: Figure S3c). Thus, we considered the rate of 250 rpm and duration of 30 s to be optimal conditions of stirring.

**Fig. 1:**
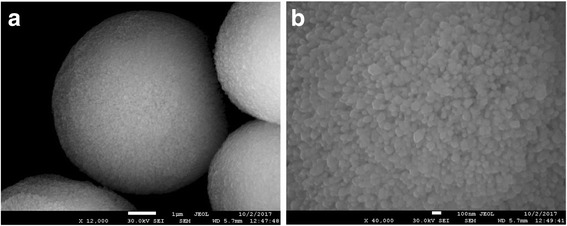
Scanning electron microphotographs of calcium carbonate microparticles (**a**) and their surface at a higher magnification (**b**)

According to scanning electron microscopy (SEM) data, the surface of the calcium carbonate microparticles was heterogeneous, characterized by porosity (Fig. 1a). At a magnification of × 40,000, it could be seen that the microparticles were, in turn, formed by smaller, submicrometer particles (Fig. 1b). Thus, the obtained microparticles had a porous structure and represented matrices which not only are suitable as substrates, but also can be used per se as delivery systems and due to their particular surface structure may be easily used as a matrice for layer-by-layer polymer deposition.

### Preparation and Characterization of Polyelectrolyte Microcapsules Encoded with Quantum Dots

**Fig. 2:**
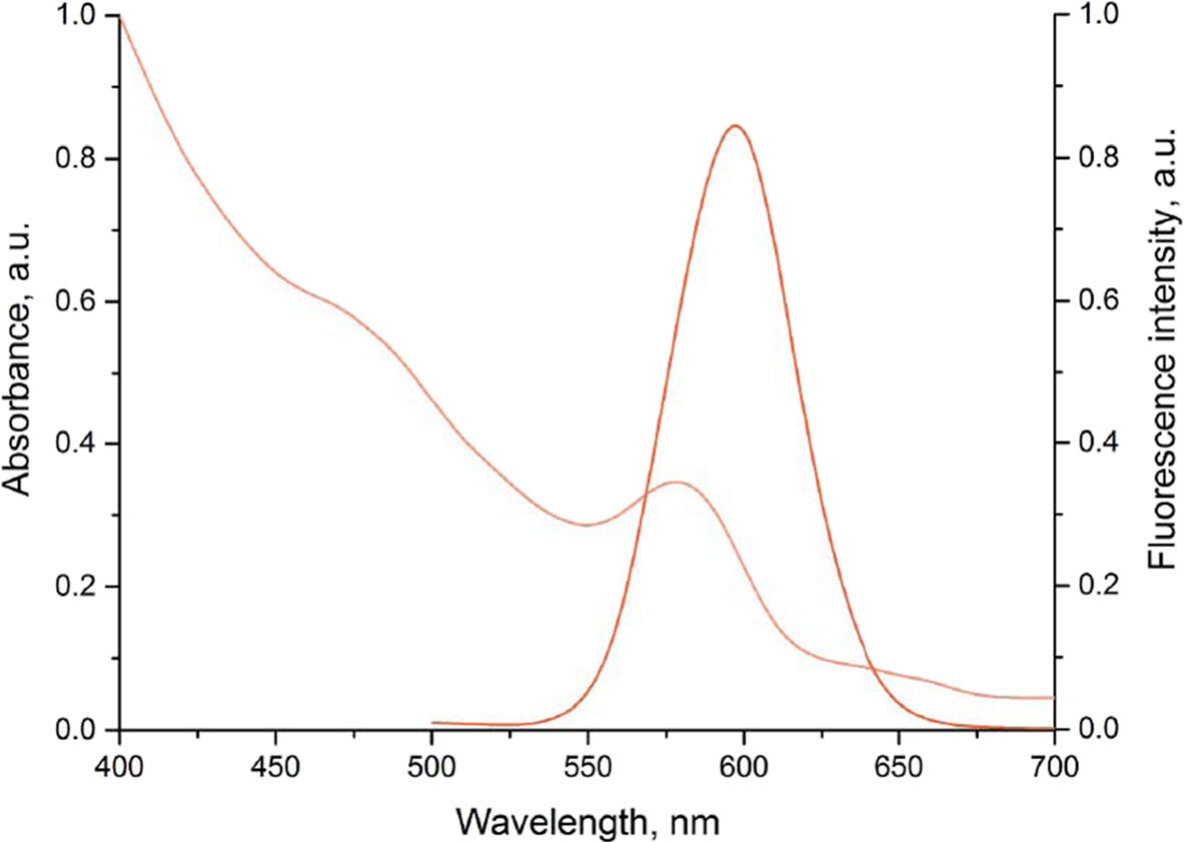
Optical characteristics of the CdSe/ZnS core/shell quantum dots solubilized with HS−(PEG)_12_−COOH ligands

The prepared water-soluble QDs were characterized by a wide absorption spectrum and a narrow fluorescence spectrum with an emission max of 590 nm (Fig. 2). The hydrodynamic diameter of these QD samples ranged from 23.96 to 28.2 nm. Additional file 1: Figure S4 shows the size distribution of the QDs stabilized with HS−(PEG)_12_−COOH. The modification of the QD surface with HS−(PEG)_12_−COOH ensured the QD stability in the water phase, as well as the surface negative charge sufficient for the effective adsorption of QDs between positively charged polyelectrolyte layers during the encoding procedure [22, 47].

The measured surface charge values of the calcium carbonate microparticle samples during each step of deposition of the polyelectrolyte layers and QDs (Table 1) confirmed that the surface charge of the original matrice, the solubilized QDs, as well as the surface recharge after each polymers deposition are sufficient for the effective absorption of each subsequent layer.

**Table 1:** Surface charge of the microparticles upon of polymer layer deposition and encoding with quantum dots. Number of each repeated measurements *n* = 5

Sample	ζ- potential, mean ± SD, mV
Surface before layering of polymers and quantum dots	
CaCO_3_ microparticles	− 6.0 ± 1.6
QD–S–(PEG)_12_–COOH	− 21.9 ± 0.4
Microparticle surface after layering of polymers and quantum dots	
Layer 1, PAH	+ 13.1 ± 0.8
Layer 2, PSS	− 17.8 ± 0.9
Layer 3, PAH	+ 12.7 ± 0.5
Layer 4, PSS	− 26.3 ± 1.0
Layer 5, PAH	+ 13.2 ± 0.6
Layer 6, QD–HS–(PEG)_12_–COOH	− 9.3 ± 0.6
Layer 7, PAH	+ 9.9 ± 0.4
Layer 8, PSS	− 16.9 ± 1.0
Layer 9, PAH	+ 11.6 ± 0.3
Layer 10, PSS	− 22.7 ± 0.8
Layer 11, PAH	+ 14.9 ± 0.6
Layer 12, PAA	− 25.3 ± 0.7
Layer 12, PAA, after removal of the core	− 19.9 ± 0.3
Layer 12, PAA, after coating with BSA	− 18.0 ± 1.6

**Fig. 3:**
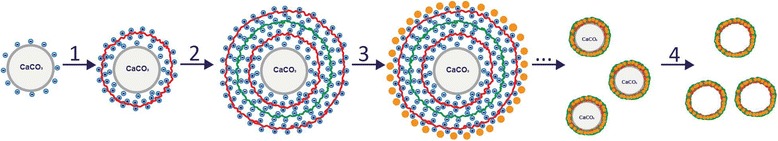
Preparation procedure for microcapsules encoded with quantum dots: formation of the layers of the polycation (1) and polyanion (2), the PAH and PSS polyelectrolytes, respectively, on the matrice surface; encoding of the resultant microparticles with quantum dots and further layer-by-layer polymers deposition (3); removal of the calcium carbonate core (4)

The intrinsic surface charge and the porous surface structure of the synthetic calcium carbonate microparticles allowed them to be used as a matrice for the oppositely charged polyelectrolyte and QD deposition (Fig. 3). Application of PAH and PSS polymers in QD-encoded polyelectrolyte polymeric microcapsules is determined by their biocompatibility and non-toxicity and also non-biodegradability that additionally helps to retain QDs within the shell. The biodegradability of the poly-l-arginine, poly-l-lysine, chitosan, alginic acid sodium salt, and dextrane sulfate which are also widely used in polyelectrolyte microcapsules formation will induce the QD diffusion out of the polymeric membrane that should result in decrease of the fluorescent properties of the microparticles [3, 11, 39, 48–52]. The PAH polycation and PSS polyanion used in this study contain, respectively, amine and sulfate groups ensuring electrostatic interaction between the polymer layers, that results in the formation of interpolymer complexes [16, 25, 36, 37]. The choice of the first polymer layer was determined by the surface charge value of the synthesized calcium carbonate microparticles.

**Fig. 4:**
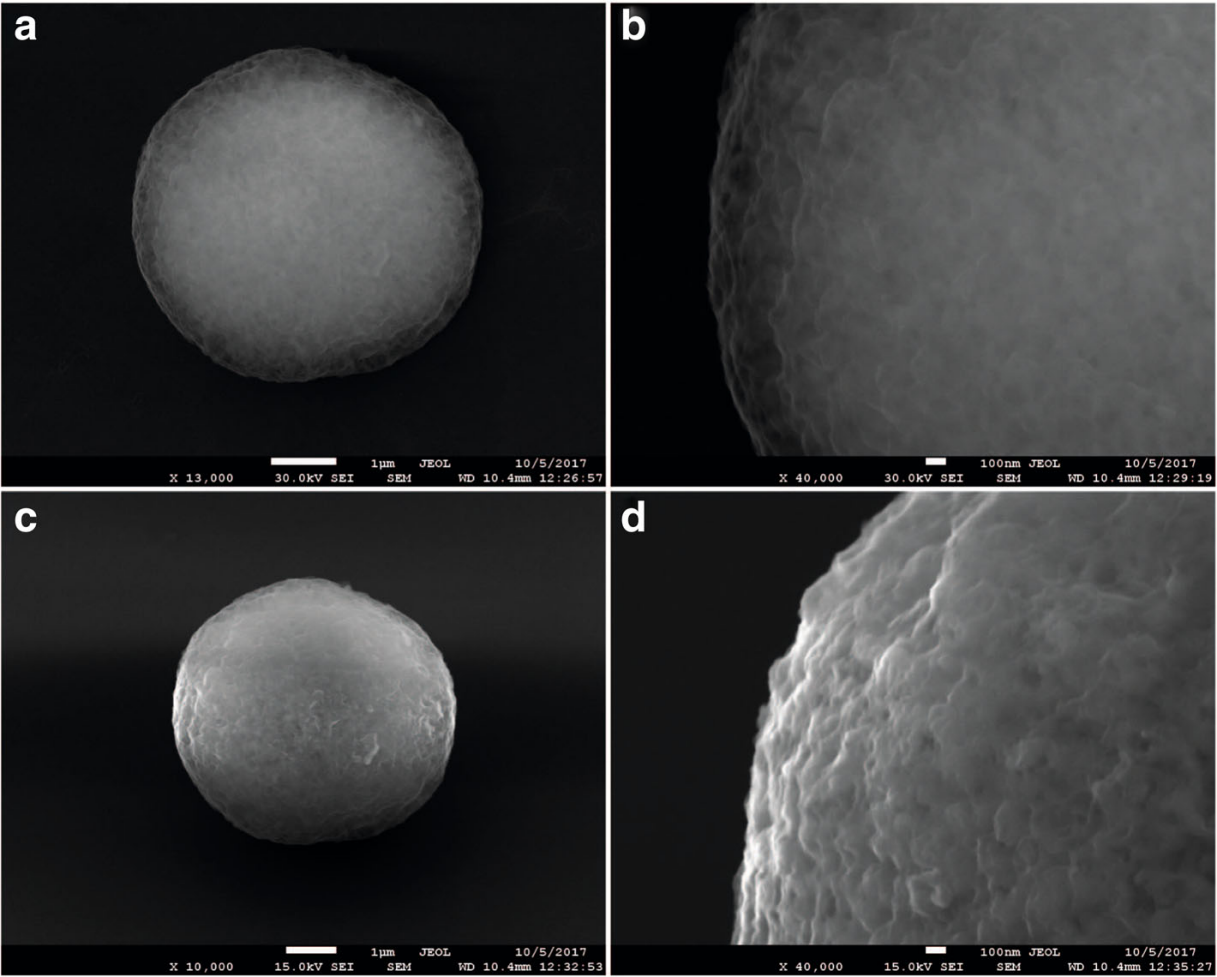
Scanning electron microscopy images of calcium carbonate microparticles after application of four (**a**, **b**) and ten (**c**, **d**) polyelectrolyte layers

Figure 4 shows SEM images of the stages of polymer shell formation on the substrate surface. As seen in the microphotographs, the microparticles contained a core of calcium carbonate grains and a shell, which became more distinct with increasing number of the adsorbed polymer layers. The surface of the microparticles coated with the polyelectrolyte layers followed the shape of the substrate with its characteristic homogeneity, which suggested that it was also porous (Fig. 4a, b) [44]. As the polymer shell became thicker, the microparticle surface became more even and smooth (Fig. 4c, d).

The final step of preparation QD-encoded polyelectrolyte microcapsules included the removal of the calcium carbonate core and formation of the ultimate structure of the microcapsules. To dissolve the matrice consisting of calcium carbonate grains, the microparticles were washed with EDTA. EDTA was used primarily because it forms water-soluble complexes upon interaction with salts of divalent metals, including calcium, and its low molecular weight ensures permeability of the polyelectrolyte shell for EDTA and its complexation with Ca^2+^. This leads to the dissolution of the core of the polyelectrolyte particles and formation of a hollow structure [45, 46].

**Fig. 5:**
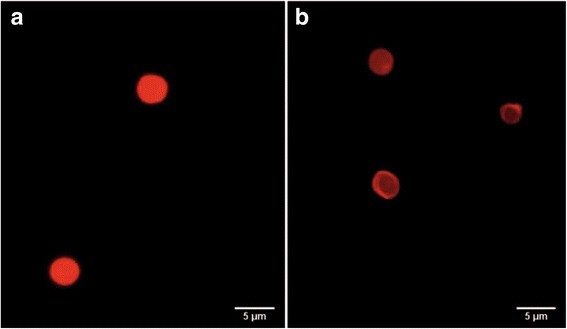
Fluorescence microscopy images of calcium carbonate microparticles coated with polyelectrolyte and encoded with quantum dot (**a**) and polyelectrolyte microcapsules obtained from them (**b**)

**Fig. 6:**
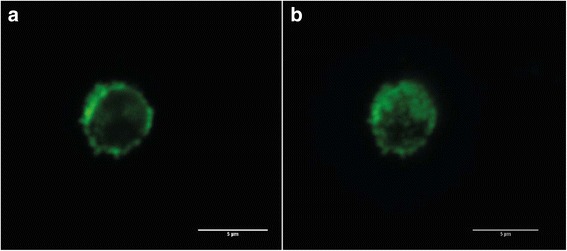
Confocal microscopy images of the polyelectrolyte microcapsules encoded with quantum dots and coated by BSA: cross-sections of the microcapsules (**a**); 3D projection of a single polyelectrolyte microcapsule (**b**)

The QD-encoded microparticles and polyelectrolyte microcapsules obtained in our study had a spherical or nearly spherical shape and a size from 3.8 to 6.5 μm (Fig. 5). Analysis of the morphology and structure of the microparticles and microcapsules in the fluorescent mode showed cavities within the polyelectrolyte microcapsules, as evident by their higher transparency compared to the microparticles (Fig. 5b). This demonstrated that the procedure of core dissolution with EDTA was effective. The confocal microscopy data also showed the hollow structure of the obtained fluorescent polyelectrolyte microcapsules (Fig. 6). These microcapsules could be distinguished as single particles (Fig. 6a, b) which can be characterized as spherical particles with rough surface due to their surface modification by BSA coating.

### Estimation of Encoding Efficiency

**Fig. 7:**
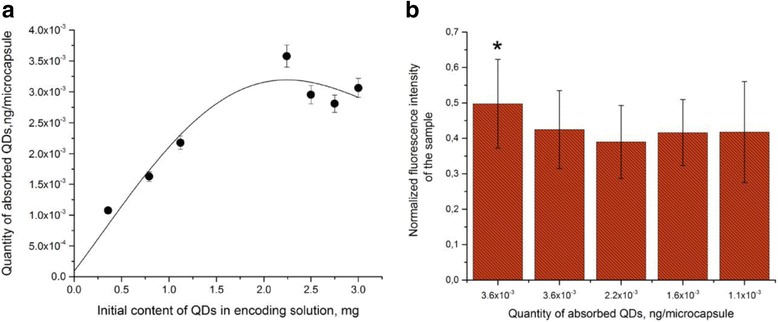
Estimation of the efficiency of encoding of microparticles with different amounts of quantum dots (**a**) and their fluorescence characteristics (**b**). Asterisk indicates significant difference of QD-encoded microbeads from the QD-encoded microcapsules (*p* < 0.05)

The efficiency of encoding was estimated by the amount of QDs adsorbed on the positively charged polymer surface of the microparticle. Estimation of the amounts of QDs in the original solution used for microparticle encoding and in the supernatant before and after incubation of microparticles with the QD solution showed that the number of QDs bound to the microparticle surface linearly increased with increasing QD content in the reaction mixture from 0.36 to 2.241 mg (Fig. 7a). Further increase in the amount of QDs in the solution led to a decrease in the number of adsorbed QDs. This may have resulted from an insufficient density of the positive charge determined by amine groups of PAH on the microparticle surface because of an excessive amount of QDs and the resultant saturation of the surface with them. Apparently, the QDs were adsorbed more efficiently if their amount was smaller than 2.241 mg owing to sterically favorable conditions and less interference with one another’s attachment. The pattern of the dependence of the encoding efficiency on the QD content of the solution used for the encoding agrees with our earlier data [22].

Estimation of the optical characteristics of the obtained polyelectrolyte microcapsules is an essential stage of the assessment of encoding efficiency. Its results show how suitable the given technique is for fabrication of imaging agents based on QD-encoded polyelectrolyte microcapsules.

Figure 7b shows the fluorescence intensities of the microparticles and microcapsules measured as the mean normalized intensity of gray color as dependent on the amount of QDs used for encoding them. As seen from the figure, the fluorescence intensity of the encoded microparticles was higher than that of the microcapsules obtained from them. At the same time, the microcapsules encoded with different amounts of QDs did not differ significantly from one another in fluorescence intensity (*p* > 0.05). Despite some decrease in the fluorescence intensity of the microcapsules, their encoding with the amount of QDs indicated above ensures a contrast sufficient for effective imaging.

### Quantum Dot Fluorescence Lifetime within Encoded Polyelectrolyte Microcapsules

As noted above, the fluorescence intensity of the polyelectrolyte microcapsules was decreased compared to the microparticles used for their fabrication and encoded with the same QDs. Therefore, we have estimated the fluorescence lifetimes of both the original QDs and the same QDs embedded in the microparticles or the polymeric walls of the microcapsules.

**Fig. 8:**
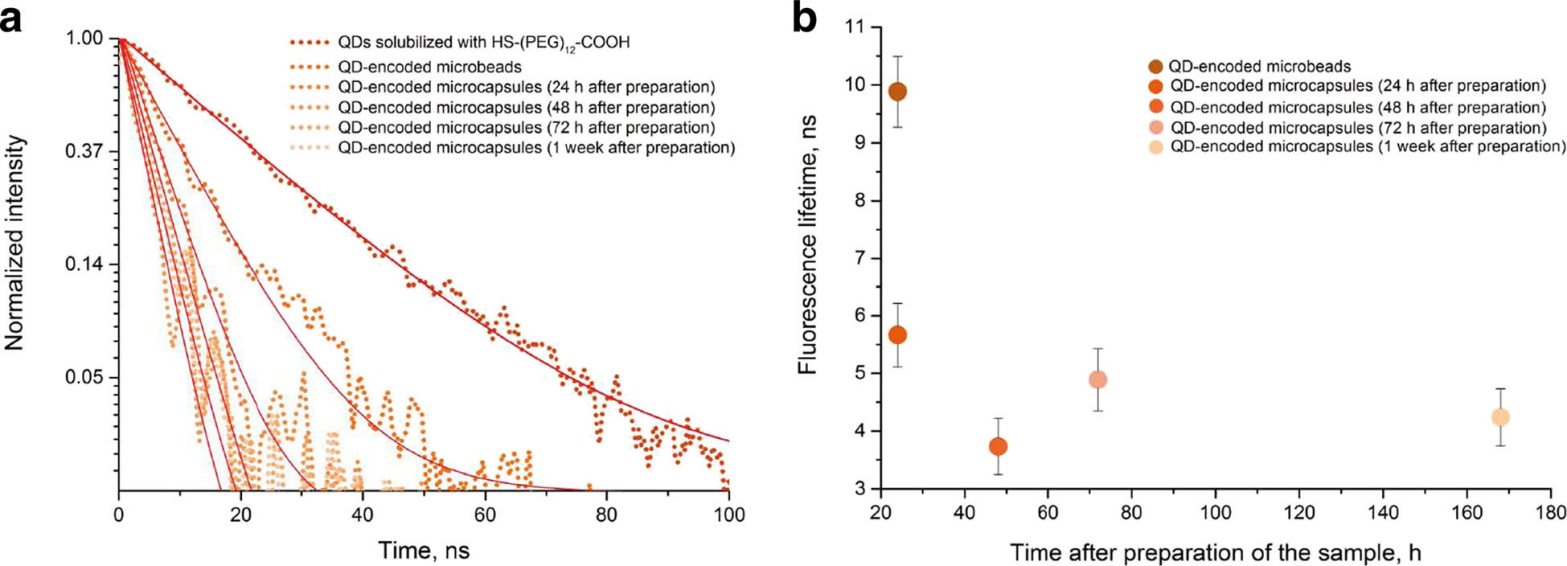
Fluorescence lifetimes of the solubilized CdSe/ZnS quantum dots with a fluorescence peak at 590 nm incorporated in the fabricated microparticles and microcapsules

The QD fluorescence kinetic curves (Fig. 8) are characterized by monoexponential dependence of fluorescent intensity on time according to the following equation:

$$ I(t)={A}_1\bullet {e}^{-x/{t}_1}, $$

where *I*(*t*) is the intensity of QD fluorescence in response to excitation pulses and *A*_1_, *х*, and *t*_1_ are parameters describing the change in the intensity with time.

We determined the fluorescence lifetime for each sample (Table 2). The original solubilized QDs had the longest fluorescence lifetime; it was decreased after the QDs were adsorbed onto the microparticles and incorporated into the structure of the polymer shell. This may have resulted from interaction between the QDs and PAH, as we found earlier [22]. In the case of microcapsules, the QD fluorescence lifetime tended to further decrease compared to the QDs within the microparticles from which the microcapsules were fabricated. A possible cause of this decrease was a technological factor entailed in the fabrication of microcapsules, namely, the dissolution of the core and the related increase in the necessary number of washings.

**Table 2:** Quantum dot fluorescence lifetime data

Sample	Lifetime, ns^*^
Solubilized QDs	21.09
QD-encoded microparticles	9.88
QD-encoded microcapsules (24 h after fabrication)	5.99^**,***^
QD-encoded microcapsules (48 h after fabrication)	3.82^**^
QD-encoded microcapsules (72 h after fabrication)	5.12
QD-encoded microcapsules (1 week after fabrication)	4.39^***^

*QD,* quantum dot

*The microparticles and microcapsules significantly differ from the solubilized quantum dots in fluorescence lifetime (*p* < 0.05)

**, ***Significant differences between the samples of microcapsules in fluorescence lifetime (*p* < 0.05)

It should be noted, that the fluorescence lifetime also tended to decrease with time after the microcapsule fabrication. However, since 48 h after the microcapsule fabrication, further changes in the mean fluorescence lifetime were insignificant. The fluorescence decay was apparently caused by a decrease in the fluorescence quantum yield of QD embedded in the shells of the capsules. This effect is likely to have resulted from the change in the distribution of the electron potential and the geometric rearrangement of QDs in the inner layers of the polymer shell after the core removal that increased the probability of nonradiative recombination due to the charge transfer to between neighboring QDs or between QDs and polymer molecules [22].

### Interaction of Quantum Dot*-*Encoded Polyelectrolyte Microcapsules with Phagocytic Cells In Vitro

We used confocal microscopy to analyze the interaction of QD-encoded polyelectrolyte microcapsules with live phagocytic cells, their uptake by cells, and the possibility of cell labeling. The murine alveolar macrophage (MH-S) cells were used as a model, because of the capability of these cells to phagocytize xenogenic objects.

**Fig. 9:**
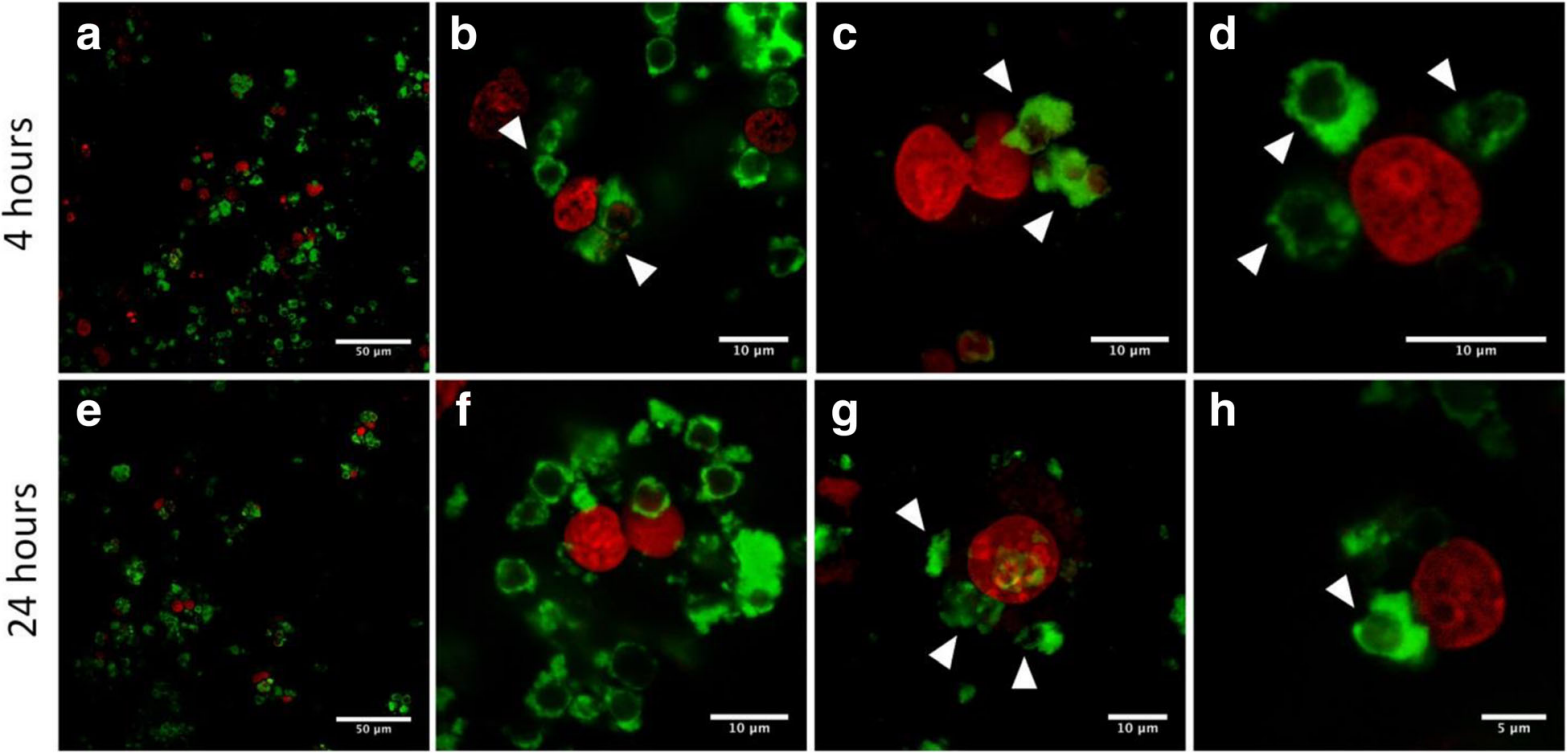
Confocal images of the MH-S cells treated with the QD-encoded polyelectrolyte microcapsules coated with BSA. The upper row shows the images of the samples after 4 h of short-term incubation; the microcapsules are shown by white arrows (**a**–**d**). The lower row demonstrates the images of the samples after 24 h of long-term incubation; the microcapsules are shown by white arrows (**e**–**h**). The nuclei of the macrophages were counterstained with far-red DNA stain DRAQ5

The MH-S cells were treated with approximately 1.2×10^6^ of QD-encoded microcapsules by short-term (4 h) or long-term (24 h) incubations. We observed signs of primary uptake of the microcapsules in both cases: after the short- and (Fig. 9a–d) long-term incubation (Fig. 9e–h). Polyelectrolyte microcapsules or their conglomerates could be seen in green color. The microcapsules could be clearly distinguished both in the external environment of cells and inside the MH-S cells that could be evidenced by the distance between the microcapsules and nuclei of the MH-S cells which were stained by far-red DNA stain DRAQ5 and can be seen as red spherical shaped objects at all the microphotographs. As individual microcapsules and as their conglomerates were detected to undergo the uptake process. The fact that microcapsules were located in internal cell environment or at least attached to the surface of the macrophages is confirmed by clearly distinguished short distances between the nuclei and the polyelectrolyte microcapsules (Fig. 9b, d, g, h). In Fig. 9g, residually stained boundaries of the macrophage cytoplasmic membranes are distinctly seen, which indicates effective uptake, and well-discernible microcapsules are easy to detect to be attached on their surface either within the cells.

During the short-term incubation, single microcapsules were found to be phagocytized by MH-S cells prior to conglomerates of the microparticles. While after long-term incubation (24 h), the amount of the conglomerates of the polyelectrolyte microcapsules undergoing the uptake process and located inside cells or at least attached to the cell surface was more significant than after short-term incubation. Thus, the polyelectrolyte microcapsules obtained in this study used as promising tools for imaging and tracking of live cells.

## Conclusions

Our study has demonstrated the feasibility of fabrication of stable QD-encoded polyelectrolyte microcapsules with optimized fluorescence characteristics and a narrow size distribution. The technique for incorporation of water-soluble and stabilized with three-functional polyethylene glycol derivatives core/shell QDs into the polymer wall of the microcapsules and detailed characterization at each stage of experimental procedure ensured efficient fluorescent encoding of microcapsules. The efficient intracellular uptake of developed QD-encoded microcapsules by murine macrophages was demonstrated, thus confirming the possibility of efficient use of developed system for live cell imaging and visualization of microcapsules transportation and delivery within the living cells.

## Additional file

Additional file 1: Figure S1. Schematic diagram of a theranostic agent based on polyelectrolyte microcapsules.** Figure S2.** Size distributions of calcium carbonate microparticles obtained at stirring rates of 250 (**a**), 500 (**b**), and 750-rpm (**c**). The stirring duration was 30 s in all cases. The size distribution diagrams are based on the measurements of individual microparticles (*n* = 350). The differences between samples **a**, **b**, and **c** are significant (*p* < < 0.05). **Figure S3.** Size distributions of calcium carbonate microparticles obtained at stirring durations of 15 (**a**), 30 (**b**), and 60 s (**c**). The stirring rate was 250 rpm in all cases. The size distribution diagrams are based on the measurements of individual microparticles (*n* = 350). The differences between samples **a**, **b**, and **c** are significant (*p* < < 0.05). **Figure S4.** Size distribution of the solubilized quantum dots as estimated by the volume occupied by the particles (**a**), the number of the particles (**b**), or the light scattering intensity (**c**). (DOCX 457 kb)

## Abbreviations

EDTA: Disodium ethylenediaminetetraacetat
PAA: Polyacrylic acid
PAH: Polycation poly(allylamine hydrochloride)
PEG: Polyethylene glycol
PSS: Polyanion poly(sodium 4-styrenesulfonate)
QD: Quantum dot
RPMI medium: Roswell Park Memorial Institute medium
SEM: Scanning electron microscopy
TOPO: Trioctylphosphine oxide

## References

[1] Xiong R, Soenen SJ, Braeckmans K, Skirtach AG (2013). Towards theranostic multicompartment microcapsules: in-situ diagnostics and laser-induced treatment. Theranostics.

[2] Namdee K, Thompson AJ, Golinski A, Mocherla S, Bouis D, Eniola-Adefeso O (2014). In vivo evaluation of vascular-targeted spheroidal microparticles for imaging and drug delivery application in atherosclerosis. Atherosclerosis.

[3] De Koker S, De Geest BG, Cuvelier C, Ferdinande L, Deckers W, Hennink WE (2007). In vivo cellular uptake, degradation, and biocompatibility of polyelectrolyte microcapsules. Adv Funct Mater.

[4] Yong KT, Wang Y, Roy I, Rui H, Swihart MT, Law WC (2012). Preparation of quantum dot/drug nanoparticle formulations for traceable targeted delivery and therapy. Theranostics.

[5] Yu W, Chen Y, Mao Z (2016). Hollow polyelectrolyte microcapsules as advanced drug delivery carriers. J Nanosci Nanotechnol.

[6] Caldorera-Moore ME, Liechty WB, Peppas NA (2012). Responsive theranostic systems: integration of diagnostic imaging agents and responsive controlled release drug delivery carriers. Acc Chem Res.

[7] De Geest BG, Skirtach AG, Sukhorukov GB, Demeester J, De Smedt SC, Hennink WE (2008). Stimuli-responsive polyelectrolyte microcapsules for biomedical applications. Am Chem Soc Polym Prepr Div Polym Chem.

[8] Volodkin DV, Petrov AI, Prevot M, Sukhorukov GB (2004). Matrix polyelectrolyte microcapsules: new system for macromolecule encapsulation. Langmuir.

[9] Carregal-Romero S, Ochs M, Parak WJ (2012). Nanoparticle-functionalized microcapsules for in vitro delivery and sensing. Nano.

[10] Parakhonskiy BV, Yashchenok AM, Konrad M, Skirtach AG (2014). Colloidal micro- and nano-particles as templates for polyelectrolyte multilayer capsules. Adv Colloid Interf Sci.

[11] De Geest BG, Vandenbroucke RE, Guenther AM, Sukhorukov GB, Hennink WE, Sanders NN (2006). Intracellularly degradable polyelectrolyte microcapsules. Adv Mater.

[12] Tan C, Selig MJ, Lee MC, Abbaspourrad A (2018). Polyelectrolyte microcapsules built on CaCO_3_ scaffolds for the integration, encapsulation, and controlled release of copigmented anthocyanins. Food Chem.

[13] Salomäki M, Vinokurov IA, Kankare J (2005). Effect of temperature on the buildup of polyelectrolyte multilayers. Langmuir.

[14] Das BP, Tsianou M (2017). From polyelectrolyte complexes to polyelectrolyte multilayers: electrostatic assembly, nanostructure, dynamics, and functional properties. Adv Colloid Interf Sci.

[15] Antipina MN, Sukhorukov GB (2011). Remote control over guidance and release properties of composite polyelectrolyte based capsules. Adv Drug Deliv Rev.

[16] D́jugnat C, Sukhorukov GB (2004). pH-responsive properties of hollow polyelectrolyte microcapsules templated on various cores. Langmuir.

[17] Del Mercato LL, Rivera-Gil P, Abbasi AZ, Ochs M, Ganas C, Zins I (2010). LbL multilayer capsules: recent progress and future outlook for their use in life sciences. Nano.

[18] Song X, Li H, Tong W, Gao C (2013). Fabrication of triple-labeled polyelectrolyte microcapsules for localized ratiometric pH sensing. J Colloid Interface Sci.

[19] Zhao Q, Li B (2008). pH-controlled drug loading and release from biodegradable microcapsules. Nanomedicine Nanotechnology Biol Med.

[20] Sukhanova A, Nabiev I (2008). Fluorescent nanocrystal quantum dots as medical diagnostic tools. Expert Opin Med Diagn.

[21] Bilan R, Sukhanova A, Nabiev I (2016). Quantum dot-based nanotools for bioimaging, diagnostics, and drug delivery. Chembiochem.

[22] Bilan RS, Krivenkov VA, Berestovoy MA, Efimov AE, Agapov II, Samokhvalov PS (2017). Engineering of optically encoded microparticles with FRET-free spatially separated quantum-dot layers for multiplexed assays. ChemPhysChem.

[23] Sukhanova A, Susha AS, Bek A, Mayilo S, Rogach AL, Feldmann J (2007). Nanocrystal-encoded fluorescent microparticles for proteomics: antibody profiling and diagnostics of autoimmune diseases. Nano Lett.

[24] Resch-Genger U, Grabolle M, Cavaliere-Jaricot S, Nitschke R, Nann T (2008). Quantum dots versus organic dyes as fluorescent labels. Nat Methods.

[25] Gao H, Sapelkin AV, Titirici MM, Sukhorukov GB (2016). In situ synthesis of fluorescent carbon dots/polyelectrolyte nanocomposite microcapsules with reduced permeability and ultrasound sensitivity. ACS Nano.

[26] Voronin DV, Sindeeva O, Kurochkin M, Mayorova O, Fedosov I, Semyachkina-Glushkovskaya O (2017). In vitro and in vivo visualization and trapping of fluorescent magnetic microcapsules in a bloodstream. ACS Appl Mater Interfaces.

[27] Lengert E, Saveleva M, Abalymov A, Atkin V, Wuytens PC, Kamyshinsky R (2017). Silver alginate hydrogel micro- and nanocontainers for theranostics: synthesis, encapsulation, remote release, and detection. ACS Appl Mater Interfaces.

[28] Talapin DV, Rogach AL, Kornowski A, Haase M, Weller H (2001). Highly luminescent monodisperse CdSe and CdSe/ZnS nanocrystals synthesized in a hexadecylamine−trioctylphosphine oxide−trioctylphospine mixture. Nano Lett.

[29] Sayevich V, Guhrenz C, Dzhagan VM, Sin M, Cai B, Borchardt L (2017). Hybrid N-butylamine-based ligands for switching the colloidal solubility and regimentation of inorganic-capped nanocrystals. ACS Nano.

[30] Guhrenz C, Sayevich V, Weigert F, Hollinger E, Resch-genger U, Gaponik N (2017). Transfer of inorganic-capped nanocrystals into aqueous media. J Phys Chem Lett.

[31] Brazhnik K, Sokolova Z, Baryshnikova M, Bilan R, Efimov A, Nabiev I (2015). Quantum dot-based lab-on-a-bead system for multiplexed detection of free and total prostate-specific antigens in clinical human serum samples. Nanomedicine: NBM.

[32] Sukhanova A, Even-Desrumeaux K, Kisserli A, Tabary T, Reveil B, Millot JM (2012). Oriented conjugates of single-domain antibodies and quantum dots: toward a new generation of ultrasmall diagnostic nanoprobes. Nanomedicine: NBM.

[33] Parakhonskiy B, Zyuzin MV, Yashchenok A, Romero SC, Rejman J, Möhwald H (2015). The influence of the size and aspect ratio of anisotropic, porous CaCO3 particles on their uptake by cells. J Nanobiotechnol.

[34] Yashchenok A, Parakhonskiy B, Donatan S, Kohler D, Skirtach A, Möhwald H (2013). Polyelectrolyte multilayer microcapsules templated on spherical, elliptical and square calcium carbonate particles. J Mater Chem B.

[35] Bilan R, Ametzazurra A, Brazhnik K, Escorza S, Fernández D, Uribarri M (2017). Quantum-dot-based suspension microarray for multiplex detection of lung cancer markers: preclinical validation and comparison with the Luminex xMAP® system. Sci Rep.

[36] Sukhorukov GB, Volodkin DV, Günther AM, Petrov AI, Shenoy DB, Möhwald H (2004). Porous calcium carbonate microparticles as templates for encapsulation of bioactive compounds. J Mater Chem.

[37] Schmidt S, Uhlig K, Duschl C, Volodkin D (2014). Stability and cell uptake of calcium carbonate templated insulin microparticles. Acta Biomater.

[38] Zhang Y, Ma P, Wang Y, Du J, Zhou Q, Zhu Z (2012). Biocompatibility of porous spherical calcium carbonate microparticles on Hela cells. World J Nano Sci Eng.

[39] Volodkin D (2014). CaCO_3_ templated micro-beads and -capsules for bioapplications. Adv Colloid Interf Sci.

[40] Trushina DB, Bukreeva TV, Kovalchuk MV, Antipina MN (2014). CaCO_3_ vaterite microparticles for biomedical and personal care applications. Mater Sci Eng C.

[41] Boyjoo Y, Pareek VK, Liu J (2014). Synthesis of micro and nano-sized calcium carbonate particles and their applications. J Mater Chem A.

[42] Ariga K, McShane M, Lvov YM, Ji Q, Hill JP (2011). Layer-by-layer assembly for drug delivery and related applications. Expert Opin Drug Deliv.

[43] Tan Y, Yildiz UH, Wei W, Waite JH, Miserez A (2013). Layer-by-layer polyelectrolyte deposition: a mechanism for forming biocomposite materials. Biomacromolecules.

[44] Schönhoff M, Ball V, Bausch AR, Dejugnat C, Delorme N, Glinel K (2007). Hydration and internal properties of polyelectrolyte multilayers. Colloids Surf A Physicochem Eng Asp.

[45] Antipov AA, Shchukin D, Fedutik Y, Petro AI, Sukhorukov GB, Mo H (2003) Carbonate microparticles for hollow polyelectrolyte capsules fabrication. Colloids Surfaces A Physicochem Eng Asp. 224:175–183.

[46] Tong W, Dong W, Gao C, Möhwald H (2005). Charge-controlled permeability of polyelectrolyte microcapsules. J Phys Chem B.

[47] Gaponik N (2010). Assemblies of thiol-capped nanocrystals as building blocks for use in nanotechnology. J Mater Chem.

[48] Janeesh PA, Sami H, Dhanya CR, Sivakumar S, Abraham A (2014). Biocompatibility and genotoxicity studies of polyallylamine hydrochloride nanocapsules in rats. RSC Adv.

[49] Gribova V, Auzely-Velty R, Picart C (2012). Polyelectrolyte multilayer assemblies on materials surfaces: from cell adhesion to tissue engineering. Chem Mater.

[50] Wen Y, Xu J, Li D, Liu M, Kong F, He H (2012). A novel electrochemical biosensing platform based on poly(3,4-ethylenedioxythiophene):poly(styrenesulfonate) composites. Synth Met.

[51] Boura C, Menu P, Payan E, Picart C, Voegel JC, Muller S (2003). Endothelial cells grown on thin polyelectrolyte multilayered films: an evaluation of a new versatile surface modification. Biomaterials.

[52] Gaponik N, Radtchenko IL, Gerstenberger MR, Fedutik YA, Sukhorukov GB, Rogach AL (2003). Labeling of biocompatible polymer microcapsules with near-infrared emitting nanocrystals. Nano Lett.

