# Supplementary material for: Next-Generation Theranostic Agents Based on Polyelectrolyte Microcapsules Encoded with Semiconductor Nanocrystals: Development and Functional Characterization
Source: Nanoscale Res Lett. 2018 Jan 25;13:30. doi: 10.1186/s11671-018-2447-z (PMC5785454; doi:10.1186/s11671-018-2447-z)
Supplement: Additional file 1: Figure S1. — Schematic diagram of a theranostic agent based on polyelectrolyte microcapsules. Figure S2. Size distributions of calcium carbonate microparticles obtained at stirring rates of 250 (a), 500 (b), and 750-rpm (c). The stirring duration was 30 s in all cases. The size distribution diagrams are based on the measurements of individual microparticles (n = 350). The differences between samples a, b, and c are significant (p < < 0.05). Figure S3. Size distributions of calcium carbonate microparticles obtained at stirring durations of 15 (a), 30 (b), and 60 s (c). The stirring rate was 250 rpm in all cases. The size distribution diagrams are based on the measurements of individual microparticles (n = 350). The differences between samples a, b, and c are significant (p < < 0.05). Figure S4. Size distribution of the solubilized quantum dots as estimated by the volume occupied by the particles (a), the number of the particles (b), or the light scattering intensity (c). (DOCX 457 kb) [file 11671_2018_2447_MOESM1_ESM.docx]

**Supplementary Information**

**Next-Generation Theranostic Agents Based on Polyelectrolyte Microcapsules Encoded with Semiconductor Nanocrystals: Development and Analysis of Fluorescence Characteristics**

Galina Nifontova^1^, Maria Zvaigzne^1^, Maria Baryshnikova^1,2^, Evgeny Korostylev^3^, Fernanda Ramos-Gomes^4,^, Frauke Alves^4,5^, Igor Nabiev^1,6*^ and Alyona Sukhanova^1,6*^

^1^ Laboratory of Nano-Bioengineering, National Research Nuclear University MEPhI (Moscow Engineering Physics Institute), Kashirskoye Shosse 31, 115409 Moscow, Russian Federation

^2^ N.N. Blokhin National Medical Research Center of Oncology, Kashirskoye Shosse 24, 115478 Moscow, Russian Federation

^3^ Moscow Institute of Physics and Technology (State University), Institutskiy per. 9, 141701 Dolgoprudny, Moscow Region, Russian Federation

^4^ Translational Molecular Imaging, Max-Planck-Institute of Experimental Medicine, Hermann-Rein-Str. 3, D37075 Göttingen, Germany

^5^ Clinic of Haematology and Medical Oncology, University Medical Center Göttingen, Robert-Koch-Str. 40, 37075 Göttingen, Germany

^6^ Laboratoire de Recherche en Nanosciences (LRN-EA4682), Université de Reims Champagne-Ardenne, rue Cognacq Jay 51, 51095 Reims, France

**
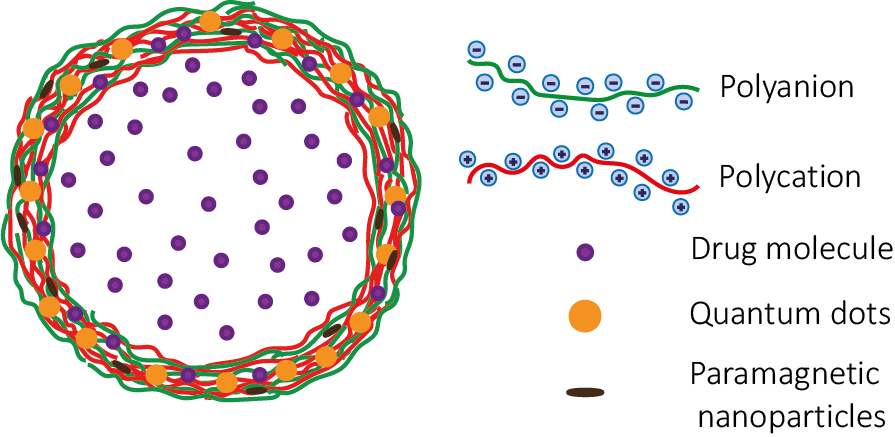
**

**Fig. S1** Schematic diagram of a theranostic agent based on polyelectrolyte microcapsules.

**
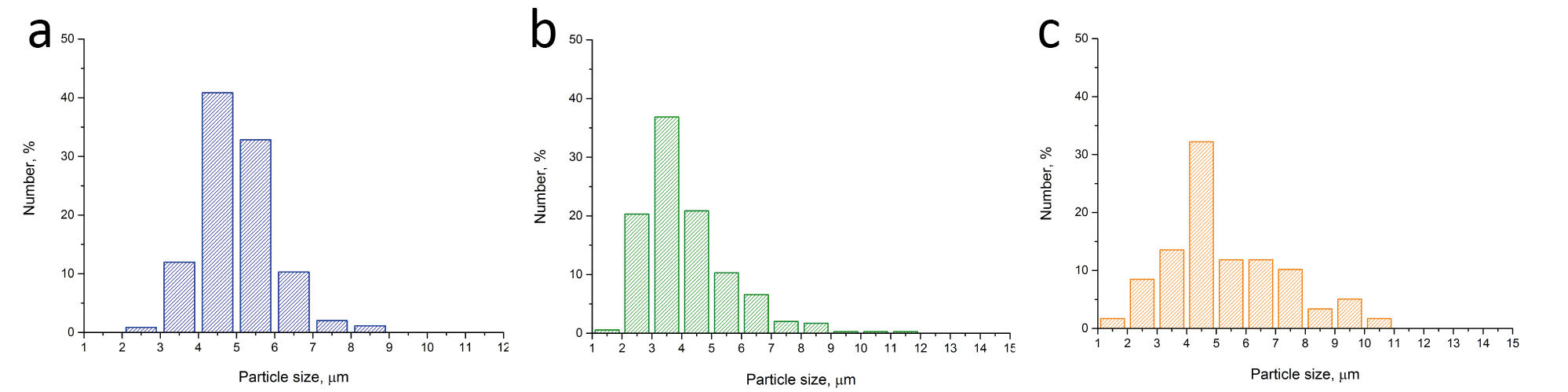
**

**Fig. S2** Size distributions of calcium carbonate microparticles obtained at stirring rates of 250 (**a**), 500 (**b**), and 750 rpm (**c**). The stirring duration was 30 s in all cases. The size distribution diagrams are based on the measurements of individual microparticles (*n* = 350). The differences between samples **a**, **b**, and **c** are significant (*p* << 0.05).

**
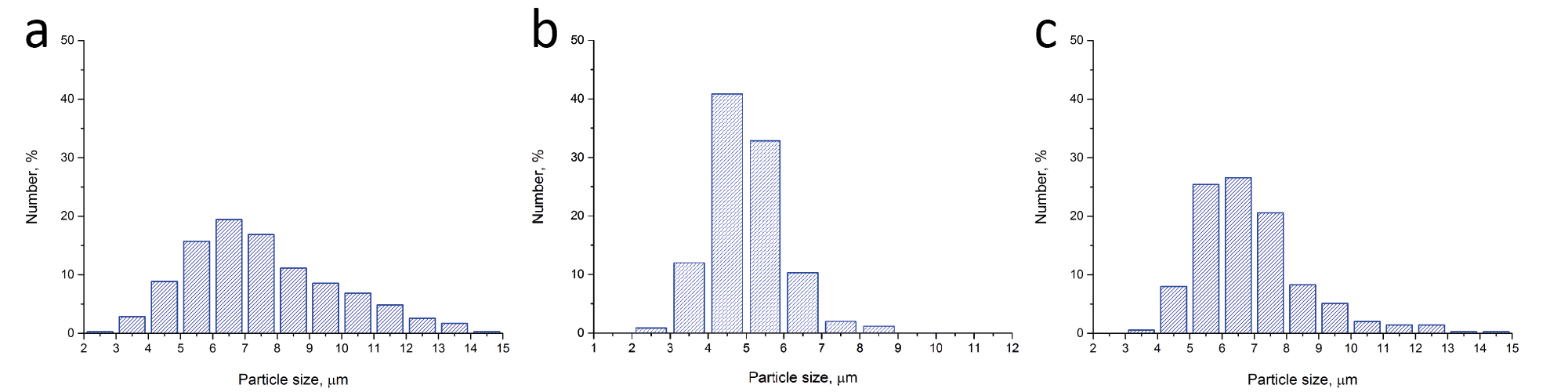
**

**Fig. S3** Size distributions of calcium carbonate microparticles obtained at stirring durations of 15 (**a**), 30 (**b**), and 60 s (**c**). The stirring rate was 250 rpm in all cases. The size distribution diagrams are based on the measurements of individual microparticles (*n* = 350). The differences between samples **a**, **b**, and **c** are significant (*p* << 0.05).

**
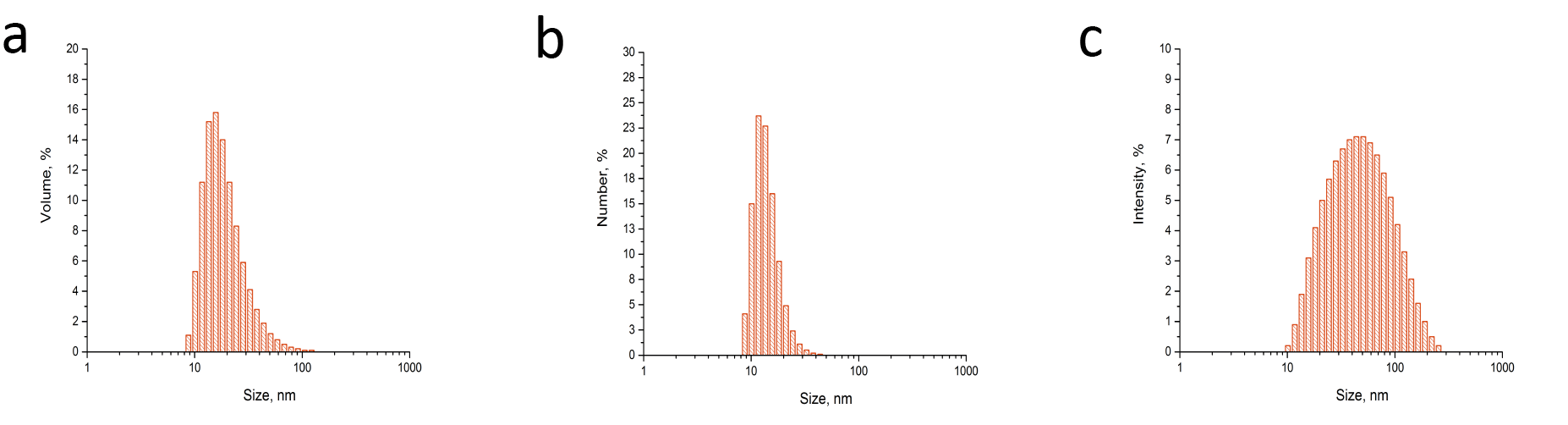
**

**Fig. S4** Size distribution of the solubilized quantum dots as estimated by the volume occupied by the particles (**a**), the number of the particles (**b**), or the light scattering intensity (**c**).
